# Stress Response of Glioblastoma Cells Mediated by miR-17-5p Targeting PTEN and the Passenger Strand miR-17-3p Targeting MDM2

**DOI:** 10.18632/oncotarget.810

**Published:** 2012-12-31

**Authors:** Haoran Li, Burton B Yang

**Affiliations:** ^1^ Sunnybrook Research Institute, Sunnybrook Health Sciences Centre, Toronto; ^2^ Department of Laboratory Medicine and Pathobiology, University of Toronto, Toronto

**Keywords:** microRNA, stem cell, miR-17, stress response, drug resistance

## Abstract

Tumor development not only destroys the homeostasis of local tissues but also the whole body, and thus the tumor cells have to face the body's defense system, a shortage of nutrition and oxygen, and chemotherapeutic drug treatment. In response to these stresses, tumor cells often alter gene expression and microRNA levels to facilitate survival. We have demonstrated that glioblastoma cells deprived of nutrition or treated with chemotherapeutics drugs expressed increased levels of miR-17. Ectopic transfection of miR-17 prolonged glioblastoma cell survival when the cells were deprived with nutrition or treated with chemotherapeutic drugs. Expression of miR-17 also promoted cell motility, invasion, and tube-like structure formation. We found that these phenotypes were the results of miR-17 targeting PTEN. As a consequence, HIF1&alpha; and VEGF were up-regulated. Ectopic expression of miR-17 was found to facilitate enrichment of stem-like tumor cells, since the cells became drug-resistant, showed increased capacity to form colonies and neurospheres, and expressed higher levels of CD133, a phenotype similar to ectopic expression of HIF1&alpha;. To further confirm the phenotypic property of stem cells, we demonstrated that glioblastoma cells transfected with miR-17 proliferated slower in different nutritional conditions by facilitating more cells staying in the G1 phase than the control cells. Finally, we demonstrated that miR-17 could repress MDM2 levels, resulting in decreased cell proliferation and drug-resistance. Our results added a new layer of functional mechanism for the well-studied miRNA miR-17.

## INTRODUCTION

Glioblastoma is the most common primary brain tumor, accounting for nearly 40 percent of all central nervous system malignancies [1]. It is characterized by an aggressive growth pattern and a resistance to conventional therapy. Despite extensive efforts, the prognosis is still very poor, with a median survival of approximately 14 months [1]. Although some advanced cancer patients can benefit from chemotherapy, residual tumors often recur soon after treatment. It is believed that a sub-population of tumor cells is not sensitive to treatment, and might be the cause of tumor relapse [2, 3]. The characteristics of these cells, however, remain largely unknown.

MicroRNAs are a group of small non-coding RNAs that are transcribed in the nuclei and transported to the cytoplasm, each precursor miRNA can be processed to produce a mature miRNA and a passenger strand [4]. Usually, the mature miRNA is the guide strand for regulation of gene expression, while the passenger strand is believed to be degraded and inactivated [5, 6]. The mature miRNAs regulate gene expression by targeting mRNAs post-transcriptionally A great deal of evidence has indicated that microRNAs play a crucial role in regulating tumor proliferation, apoptosis, angiogenesis and metastasis [7-19]. They also play important roles in development of glioblastoma [20-22]. Through the down-regulation of target proteins, microRNAs can function as oncomirs or tumor suppressors in a cell-specific context. MiR-17-92 is one of the most extensively studied clusters. This cluster and its paralogs have been shown to be associated with many malignancies such as breast cancer, liver cancer, colon cancer, lung cancer and lymphoma [23-25]. The miR-17-92 cluster has six components which share common characteristics in structure but differ in functions [26, 27]. Since each member in this cluster can mediate multiple pathways and act diversely, there is a pressing need to explore the precise role of each component. There are documented evidences that dysfunctions of microRNA are associated with the development of glioblastoma [28]. Elevated levels of miR-17 were found in glioblastoma samples and was negatively related to patients’ survival [29]. MiR-17 is also increased in glioblastoma spheroids, which are enriched in tumor initiating cells (TICs) or stem-like cells (TSCs) [30]. Thus, miR-17 has emerged as a critical regulator in mediating the cellular function of glioblastoma.

Emerging studies suggest that many stress signals are responsible for altered microRNA expression and functions [31-33]. Some microRNAs can modify gene expression by cross-talking with the tumor micro-environment, and their expression can be altered in turn by distinct stress conditions such as hypoxia, oxidative stimulation or radiation [34-42]. In response to stress, tumor cells often change gene expression to facilitate their survival [43]. For example, the up-regulation of hypoxia inducible factor-1α (HIF-1α) to adapt to oxygen and nutritional shortage is essential for inducing tumorigenesis and angiogenesis. HIF-1α is generally subjected to the negative regulation of tumor suppressors such as Von Hippel-Lindau (VHL) and phosphatase and tensin homolog (PTEN). Notably, HIF-1α is often found overexpressed in glioblastoma [44]. Our findings identified that, due to suppression of PTEN by miR-17, HIF-1α was stabilized when tumor cells were under starvation or chemotherapy, and its elevation promoted survival, motility and angiogenesis. Furthermore, HIF-1α overexpression contributed to the generation of tumor stem-like cells. Interestingly, such effects could be only achieved in stressed conditions such as serum deprivation or chemotherapeutic drug treatment, yet miR-17 reduced tumor growth by targeting murine double minute 2 (MDM2) under normal circumstances. Because the retarded proliferation rate often decreases chemo-sensitivity, miR-17-transfected cells develop resistance to chemotherapy. Hereby we show miR-17 has a dual function in glioblastoma: it suppresses tumor cell growth in normal conditions, and it also promotes tumor cell survival in unfavorable conditions. This highlights a potential mechanism in the response of tumor cells to stress and chemotherapy.

## RESULTS

### MiR-17 prolongs glioblastoma cell survival and increases cell motility

**Figure 1:**
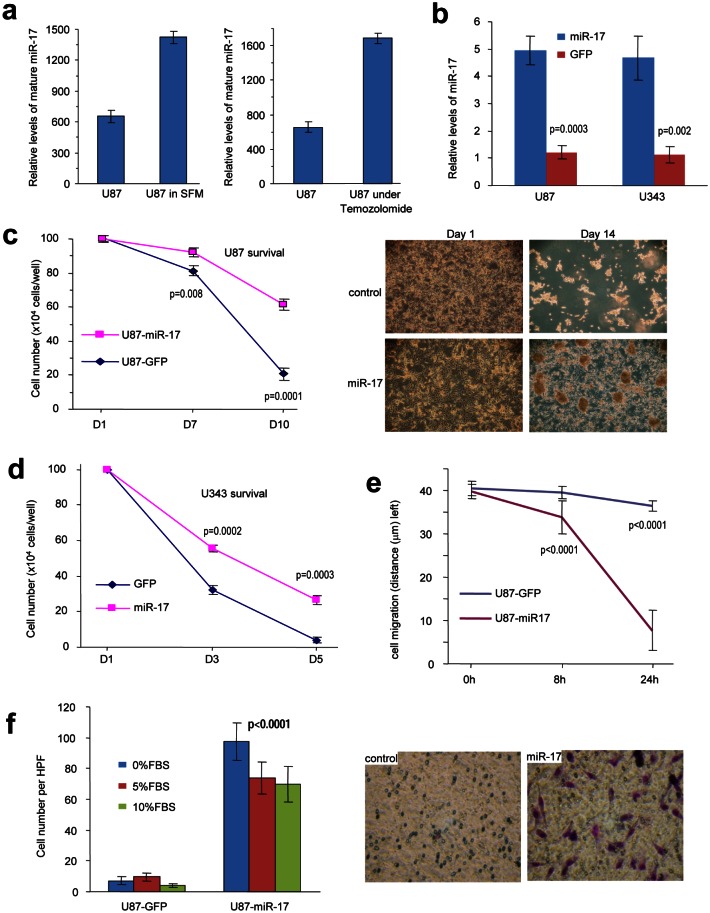
MiR-17 enhances glioblastoma cell survival, migration, and invasion (a) U87 cells were cultured in serum-free or FBS containing medium, followed by analysis of miR-17 levels. Nutrition deprivation increased miR-17 levels.U87 and U343 cells were also treated with chemo-drug Temozolomide, followed by analysis of miR-17 levels. Temozolomide treatment increased miR-17 levels. (b) Real-time PCR was performed to detect the relative mRNA levels in transfected cells. (c) U87 cells were cultured in serum-free DMEM for survival assay. The miR17-overexpression cells displayed higher ability of survival than the control cells. **p<0.001, Error bars indicate SD, n=3. (d) U343 cells were cultured in serum-free DMEM for survival assay. The miR17-overexpression cells displayed higher ability of survival than the control cells. **p<0.001, Error bars indicate SD, n=3. (e) The cells were seeded onto 6-well dishes and the monolayers were wounded with a pippette tip and cultured in 10% FBS/DMEM medium containing 2 μM mytomycin. The distances between the wounding centre and the front of the migrating cells (vertical axis) were measured for statistical analysis. **, *p<* 0.01. Error bars indicate SD (*n=*10). (f) The cells were loaded into the insert with 100 μl serum-free DMEM medium and then incubated at 37°C for 24 hours. The invasive cells were stained blue and were counted in 6 randomly selected fields under a light microscope. Expression of miR-17 promoted cell invasion. **, *p<* 0.01. Error bars indicate SD (*n=*6).

To test how glioblastoma cells responded to nutrition deprivation, we cultured U87 and U343 cells in serum-free medium or medium containing 10% FBS, followed by analysis of miR-17 levels. We found that cells expressed higher levels of miR-17-5p when nutrition was deprived than that cultured in medium containing serum (Fig 1a). The cells were also treated with the chemotherapeutic Temozolomide, followed by analysis of miR-17-5p levels. Treatment with Temozolomide promoted expression of miR-17-5p (Fig 1a).

To determine the role of miR-17 in glioblastoma cells, U87 and U343 cells were stably transfected with miR-17 expressing plasmid. Control cell lines were also established by using a plasmid without the miR-17 precursor sequence. The levels of miR-17 were detected by real-time PCR, which confirmed that the expression of miR-17-5p in the transfected cells was higher than that in the control cells (Fig 1b).

We then tested the roles of miR-17 in regulating cell survival. When U87 (Fig 1c) and U343 (Fig 1d) cells were starved in serum-free medium, there was an increased amount of survived miR-17-transfected cells compared to the control.

The abilities of survival and metastasis to large distance are often complied with tumors that are resistant to chemotherapeutic drug treatment. In order to evaluate metastasis potential, cell migration and invasiveness were measured by wound scratch assay and transwell test. In cell migration assay, miR-17-transfected cells migrated faster than the control cells (Fig 1e). Transwell test was also performed in different serum combination inside and outside of chambers. The experiments showed that more cells in the miR-17 group invaded through the membrane pores, which confirmed that over-expression of miR-17 could increase cell invasiveness (Fig 1f). In addition, our findings suggested that miR-17 conferred survival advantage to glioblastoma cells in an unfavorable condition and increased cell motility accordingly.

### MiR-17 regulates distinct response to starvation and chemotherapy

**Figure 2:**
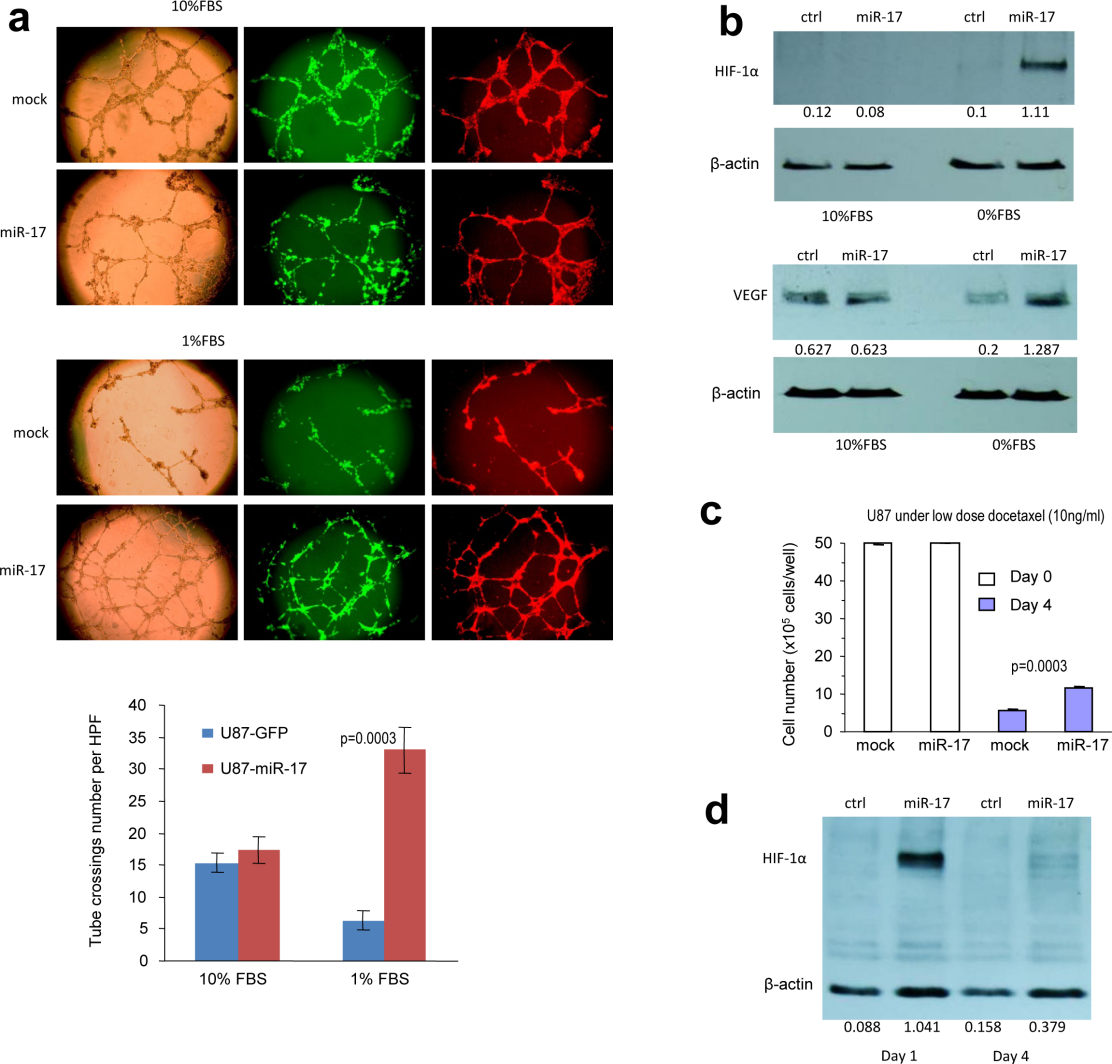
MiR-17 regulates distinct response to starvation and chemotherapy (a) The miR-17- and mock-transfected U87 cells were mixed with Ypen cells (1:1) and inoculated in Matrigel, followed by examination of formation of tube-like structures. The miR-17 expressing U87 cells formed larger complexes and longer tubes when cultured in medium containing 1% FBS, but little difference could be seen when cultured in medium containing 10% FBS. Lower, formation of the tube-like structures was quantified. (b) Expression of HIF-1α and VEGF was elevated in starved miR-17-transfected U87 cells. (c) Cell viability was analyzed in cells treated with Docetaxel. Increased survival was seen in the miR-17-transfected cells. (d) Expression of HIF-1α was elevated one day after Docetaxel treatment in the miR-17-transfected U87 cells.

Tumor expansions rely on sufficient supply of oxygen and other essential nutrients. By inducing angiogenesis, tumor cells avoid being starved and escape chemotherapy. Emerging data suggest that angiogenesis of glioblastoma involves the interactions between endothelial cells and tumor cells [45]. Tube-like structure formation is an assay widely used to study angiogenesis *in vitro*. When co-cultured with endothelial cell line, YPEN, in low serum medium, the miR-17-transfected cells induced formation of more tube-like structures than the control cells, but there was no significant difference when the cells were cultured in medium containing 10% FBS (Fig 2a). This led us to explore the expression of HIF-1α and vascular endothelial growth factor (VEGF), which are the major driving forces of vascularization. Interestingly, HIF-1α was suppressed in cells cultured in medium containing 10% FBS, but was highly expressed in the miR-17-transfected cells cultured in serum-free medium (Fig 2b). These findings suggest that HIF-1α could only be activated in starved cells expressing miR-17. HIF-1α is a downstream factor subjected to the regulation of PTEN, which highlights the possibility that miR-17 might dominate the PTEN-HIF-1α-VEGF pathway. Once PTEN was down-regulated in cells overexpressing miR-17, HIF-1α was activated in response to serum deprivation stress. HIF-1α was also reported to be negatively regulated by VHL, but we did not detect any change of VHL expression levels in either 10% FBS or serum-free conditions (Supplementary Fig S1). Given that VHL is specifically sensitive to oxygen concentration changes, PTEN-HIF-1α-VEGF pathway was shown to mainly mediate glioblastoma cells’ response to starved conditions [46]. HIF-1α activation also facilitated up-regulation of VEGF, which was elevated in miR-17-transfected cells during serum deprivation (Fig 2b).

We further found that miR-17 regulated cell response under chemotherapy. When glioblastoma cells were treated with Docetaxel, miR-17 over-expressed cells survived better than the control cells (Fig 2c). Herein, the half maximal inhibitory concentration (IC50) was calculated and applied to long-term chemotherapy. Notably, HIF-1α was induced within 1 day in the miR-17-transfected cells, which was similar to what we found during serum deprivation. However, activation of HIF-1α could not be maintained after 4 days, which might be due to reduced cell viability after prolonged treatment (Fig 2d). Since HIF-1α is involved in response to drug treatment in glioblastoma [44], our data suggested that miR-17 could confer drug resistance to the cells by regulating the PTEN/HIF-1α pathway.

### MiR-17 induces HIF-1α activation in response to stress by targeting PTEN

**Figure 3:**
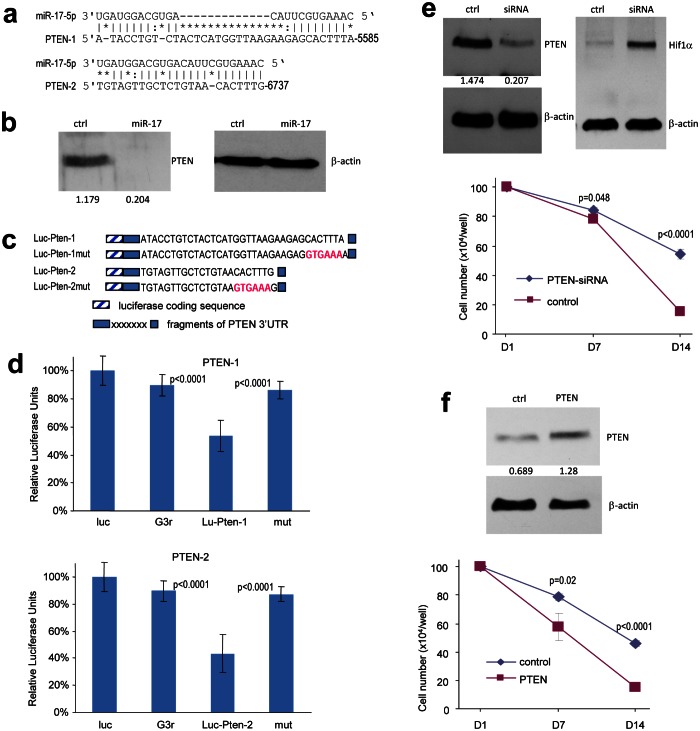
MiR-17 induces HIF-1α activation in response to stress by targeting PTEN (a) Computational analysis showed that miR-17 potentially targeted PTEN at two different sites. (b) Cell lysate prepared from miR-17- or mock-transfected U87 cells was analyzed on Western blot for PTEN expression to confirm targeting. (c) Two luciferase constructs were generated, each containing a fragment harboring the target site of miR-17, producing Luc-Pten-1 and Luc-Pten-2. Mutations were generated on the seed regions (red color), resulting in two mutant constructs Luc-Pten-1mut and Luc-Pten-2mut. (d) U87 cells were co-transfected with miR-17 and each of the luciferase reporter constructs or the mutants. The luciferase reporter vector (Luc) and the vector harboring a non-related region (G3R) were used as controls. miR-17 repressed the activity of Luc-Pten-1 and Luc-Pten-2 but had no effect on that of Luc-Pten-1mut and Luc-Pten-2mut. Error bars, SD (n=3). (e) Upper, Cell lysates prepared from U87 cells transiently transfected with siRNA targeting PTEN or a control oligo were subjected to Western blot analysis to confirm PTEN silencing. Lower, the cells were grown on 6-well tissue culture dishes. Cell survival was determined. (f) Upper, Cell lysate prepared from cells transiently transfected with PTEN expression construct or the control vector was subjected to Western blot analysis to confirm expression of the construct. Staining of β-actin from the same membrane confirmed equal loading. Lower, U87 cells stably transfected with miR-17 were transiently transfected with PTEN expression construct or the control vector and cultured for different days as indicated for survival assay. *p < 0.05. Error bars indicate SEM (n=4).

PTEN is a tumor suppressor which dominates the PTEN/HIF-1α pathway. Inactivation of PTEN often allows for the over-expression of HIF-1α, leading to cascade reactions in angiogenesis and migration. It has been reported that PTEN is a target of the miR-17-92 cluster, and indeed we detected two potential binding sites for miR-17 in PTEN 3'UTR (Fig 3a). Western blot was employed to analyze PTEN levels in the miR-17-transfected cells. Compared with the control cells, PTEN was down-regulated in cells over-expressing miR-17 (Fig 3b). The luciferase assay was then employed to determine whether miR-17 could target PTEN directly. Fragments in PTEN 3'UTR containing the binding sites of miR-17 were cloned into the pMir-report vector. Constructs with mutated binding sites were also generated to serve as controls (Fig 3c). U87 cells were co-transfected with miR-17 plasmid and one of the luciferase constructs. The experiments showed that luciferase activities were repressed when the luciferase constructs were co-transfected with miR-17 plasmid, and the inhibitory effect of miR-17 was abolished when the binding sites were mutated (Fig 3d). We then transfected U87 cells with siRNA targeting PTEN, which confirmed silencing of PTEN expression (Fig 3e, left). Down-regulation of PTEN led to increased expression of HIF-1α (Fig 3e, right), and thus prolonged the survival of the cells (Fig 3e, lower).

To confirm that PTEN played an important role in mediating miR-17 function, we transfected the miR-17-expressing cells with PTEN expression construct or a vector control. After confirming expression of the ectopic expression of PTEN (Fig 3f, upper panel), we performed cell survival assay. Ectopic expression of PTEN reversed the effect of miR-17 on cell survival (Fig 3f, lower panel).

### MiR-17 promotes the generation of tumor stem-like cells

**Figure 4:**
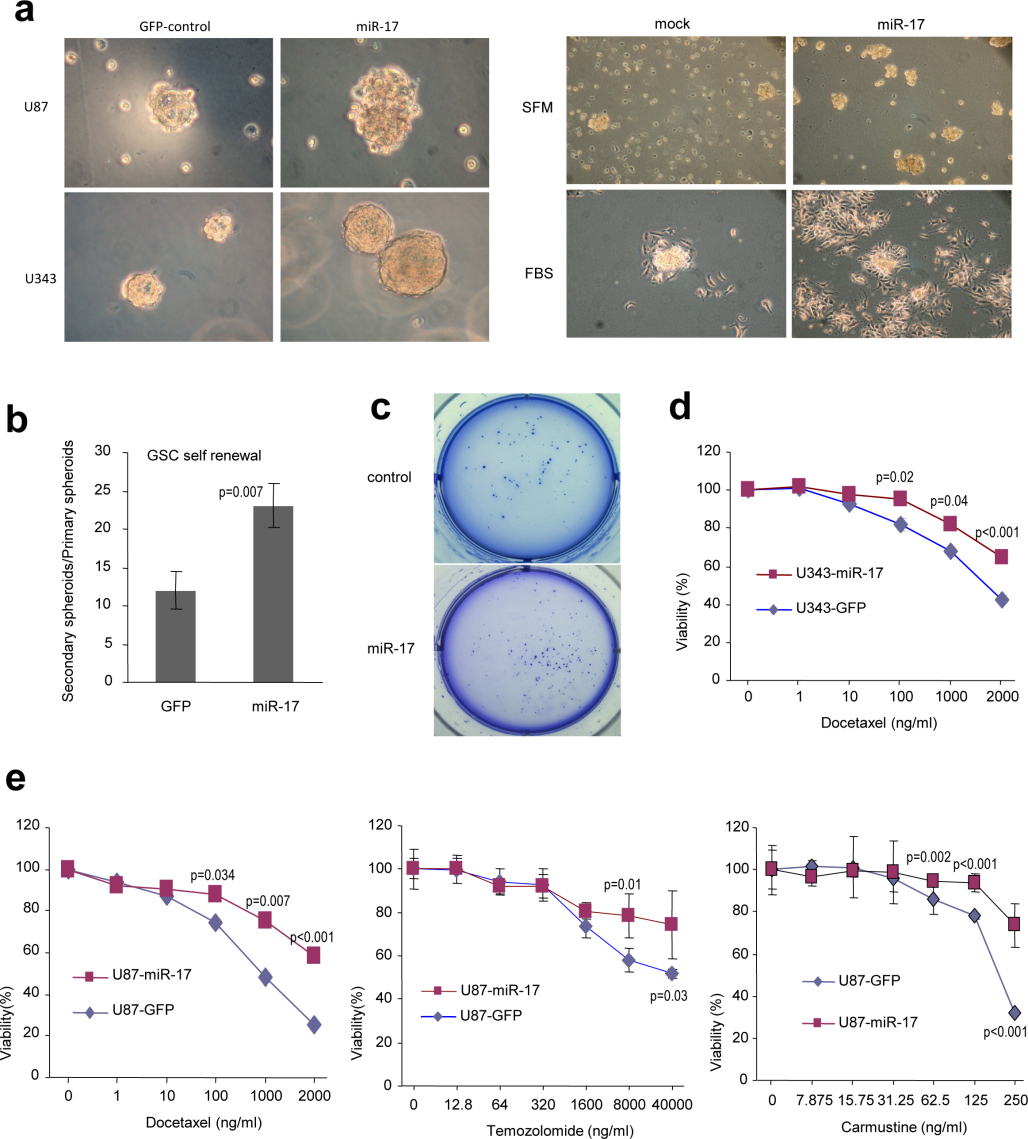
MiR-17 promotes the generation of tumor stem-like cells (a) U87 and U343 cells stably transfected with miR-17 or the control vector were cultured in serum-free medium for two weeks. Cells expressing miR-17 formed significantly larger spheres than those transfected with the mock control (left). The sphere cultures were continued to be maintained in serum-free medium, which induced extensive cell death in the control cells, but not in the miR-17-transfected cells. Addition of FBS into the cultures induced cell adhesion to the plate, displaying survivability of the spheres (right). (b) The numbers of spheres were counted and passed to new plates for continuing culture. Spheres formed in the secondary plates were divided by the numbers formed in the primary plates to evaluate the formation of spheres in the secondary plates. (c) In colony formation assays performed in soft agar, miR-17-expressing cells form more colonies with larger sizes. (d) The miR-17- and vector-transfected U343 cells were cultured and treated with Docetaxel. Sensitivities of the cells to the drug were tested. Cells transfected with miR-17 displayed resistance to Docetaxel-induced cell death. (e) The miR-17- and vector-transfected U87 cells were cultured and treated with Docetaxel, Carmustine and Temozolomide, followed by analysis of cellular viability. Cells transfected with miR-17 displayed resistance to all drugs.

It is generally thought that TSCs play a major role in tumor re-vascularization and re-aggregation, eventually leading to tumor relapse. Although the definition of TSCs is still controversial, CD133, a cell surface glycoprotein, has been used extensively as a marker of glioblastoma stem-like cells (GSC). GSCs significantly increase their number in neurospheres when cultivated in SFM containing EGF and FGF. In order to get the neurospheres, U87 and U343 cells were cultured in SFM for two weeks, and the sizes of neurospheres formed in cells overexpressing miR-17 were much larger than those formed in control cells (Fig 4a, left panel). To confirm that these spheres were alive, we continued to maintain the spheres in serum-free medium or serum-containing medium. When serum was included in the cultures, the spheres adhered to the culture plates, and this is an indication of cell survival (Fig 4a, right panel). Another prominent character of GSCs is that they can undergo self-renewal and differentiate. We thus tested the self-renewal ability of glioblastoma cells in SFM and found that the number of secondary spheres formed in cells over-expressing miR-17 was significantly higher than those formed in the control cells (Fig 4b). We then examined the tumorigenesis of glioblastoma spheroid using the colony formation assay. After the cells have been grown on agarose-containing plates for three weeks, more colonies could be seen in miR-17-transfected cells (Fig 4c).

GSCs are thought to play an important role in drug resistance. Therefore, we investigated the effects of chemotherapeutic agents on glioblastoma cells. As expected, overexpression of miR-17 facilitated cell survival after treating the U343 cells with Docetaxel (Fig 4d). We also treated the miR-17- and vector-transfected U87 cells with Docetaxel, Carmustine and Temozolomide, followed by analysis of sensitivities of the cells to these drugs. We confirmed that the cells transfected with miR-17 displayed resistance to all of these drugs (Fig 4e).

**Figure 5:**
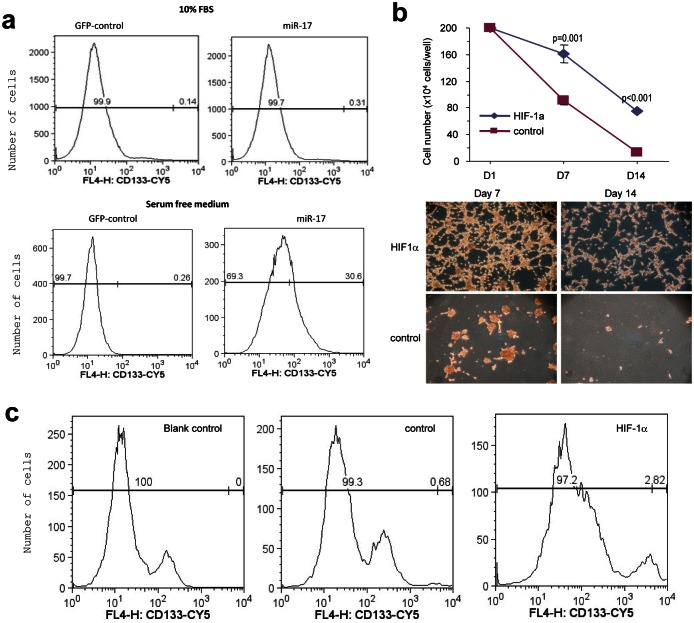
Regulation of CD133 activity by miR-17 (a) The miR-17- and vector-transfected U87 cells were subjected to flow cytometry to measured CD133 expression. CD133 expression was higher in the miR-17-transfected cells than the control cells in serum-containing medium (0.31% vs. 0.14%) or serum-free medium (30.6% vs. 0.26%). (b) Upper, U87 cells were transfected with HIF-1α or the mock vector, followed by analysis of cell survival. Transfection of HIF-1α mimicked miR-17's function in survival. Lower, Typical photos are shown. (c) The HIF-1α- and vector-transfected U87 cells were analyzed for CD133 expression. Transfection of HIF-1α increased CD133 level (2.8% vs. 0.68%).

Additionally, we examined the expression of CD133 using flow cytometry. The percentage of CD133 positive in the miR-17-transfected cells was much higher than that in the control cells (Fig 5a). Moreover, when plated in serum, these floating neurospheres could differentiate to adherent cells again. The miR-17-transfected adhesive cells still expressed higher levels of CD133 than the control cells (Fig S2).

Since we have shown that HIF-1α was up-regulated by miR-17 expression, we explored its involvement in the generation of GSCs. U87 cells were stably transfected with HIF-1α construct and plated into serum-free medium. Similar to what we observed in miR-17-transfected cells, HIF-1α over-expression increased the number of survival cells compared with the control (Fig 5b). Moreover, CD133+ ratio also increased in HIF-1α transfected cells (Fig 5c).

### MiR-17 reduces glioblastoma cell proliferation

**Figure 6:**
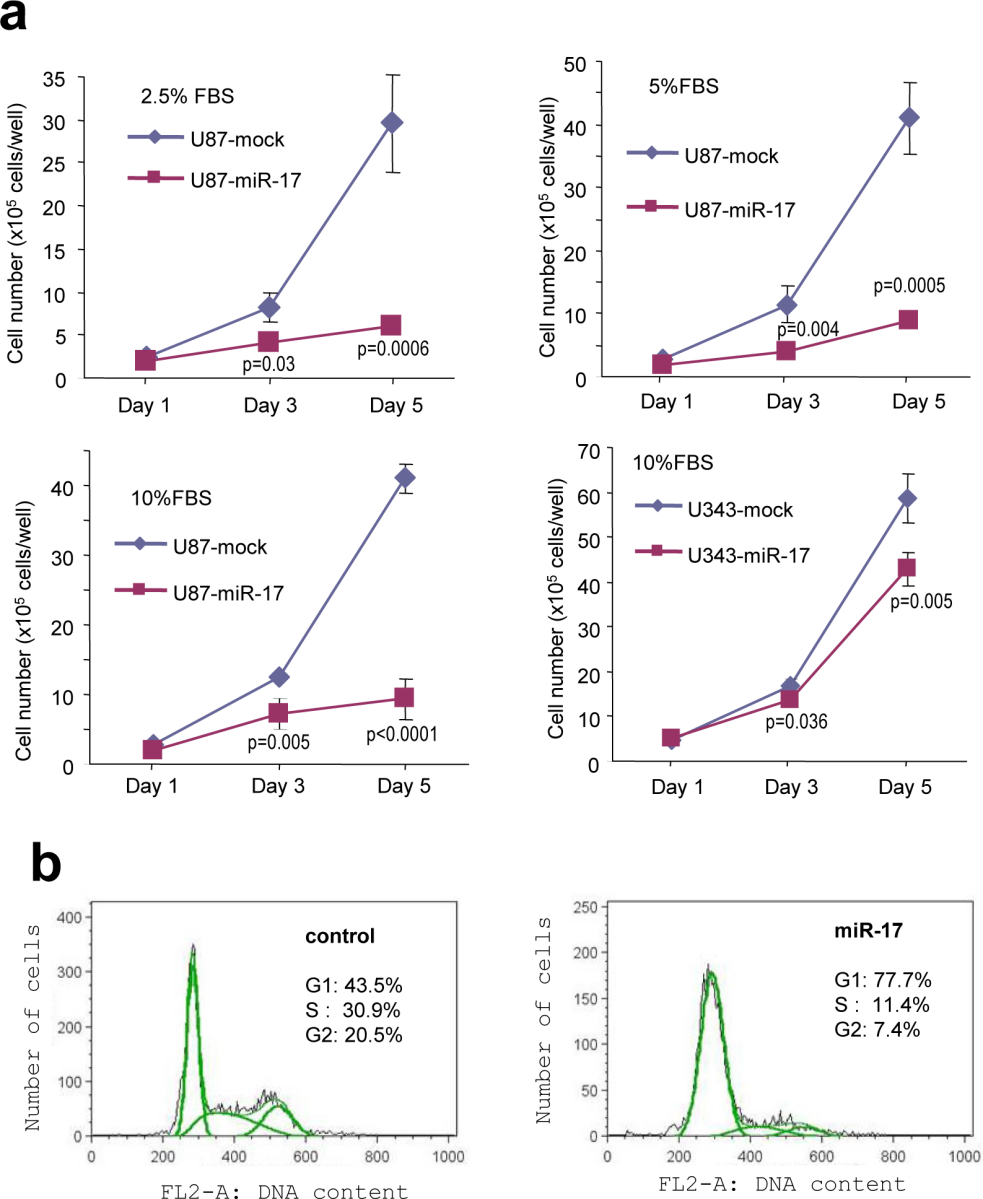
Expression of miR-17 reduces glioblastoma cell proliferation (a) Cell proliferation was inhibited in miR-17-transfected U87 and U343 cells in different fetal bovine serum (FBS) concentrations. (b) Cell cycle analysis was performed by flow cytometry, which showed that miR-17 overexpression increased the distribution of the cells in G1 phase.

In order to examine the effect of miR-17 on glioblastoma cell growth, a cell proliferation assay was performed and miR-17-overexpressing cells were found to have a significantly reduced growth rate (Fig 6a). This was in line with cell cycle analysis, in which the percentage of cells in G1 phase was much higher in miR-17-overexpressed cells as compared with the control (Fig 6b). Our findings indicated that miR-17 inhibited glioblastoma cell proliferation, which is in agreement with other studies revealing similar results on breast cancer cells [47]. Taken together, these results suggest that miR-17 suppresses glioblastoma cell growth under normal circumstances.

### MiR-17-3p targets MDM2 in glioblastoma cells

**Figure 7:**
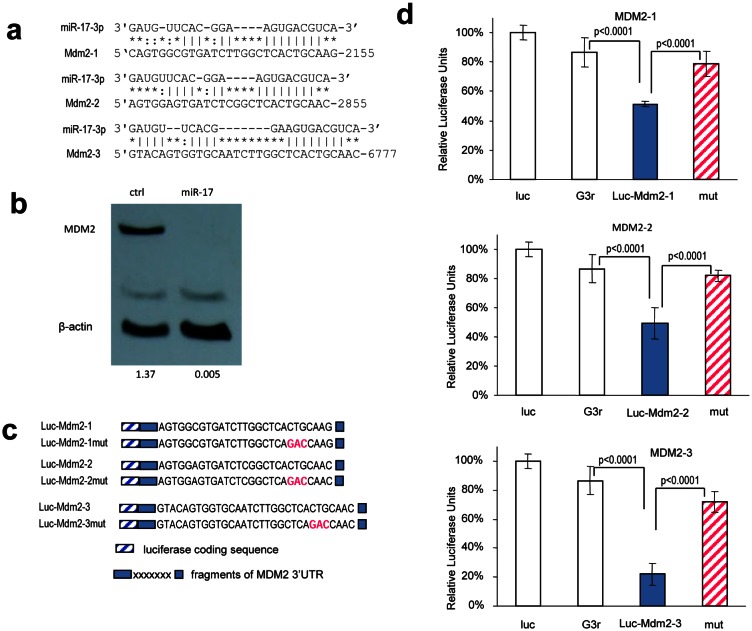
MiR-17-5p and miR-17-3p target MDM2 (a) Computational analysis showed that miR-17-5p and miR-17-3p potentially targeted MDM2 at three different sites. (b) Cell lysate prepared from miR-17- or mock-transfected U87 cells was analyzed on Western blot for MDM2 protein expression. MDM2 level was down-regulated in miR-17-transfected cells. Staining of β-actin from the same membrane confirmed equal loading. (c) Three luciferase constructs were generated, each containing a fragment harboring the target site of miR-17-3p, producing Luc-Mdm2-1, Luc-Mdm2-2, and Luc-Mdm2-3. Mutations were generated on the seed regions of each target sequence (red color), resulting in four mutant constructs Luc-Mdm2-1mut, Luc-Mdm2-2mut, and Luc-Mdm2-3mut. (d) U87 cells were co-transfected with miR-17-3p and each of the luciferase reporter constructs or the mutants. The luciferase reporter vector (Luc) and the vector harboring a non-related region (G3R) were used as controls. Asterisks indicate significant differences. Error bars, SD (n=3).

We then sought to identify the targets that mediated miR-17 suppressing glioblastoma cell growth. Taking advantage of the online databases and computational algorithms, we screened a series of genes that could promote cell proliferation. In silico analysis, MDM2 revealed three potential binding sites for miR-17-3p in its 3'UTR (Fig 7a). MDM2 is an oncogene which is highly expressed in glioblastoma and it widely participates in tumorigenesis and progression. It is thought to inhibit the activation of p53, but it can also regulate tumor cell proliferation independently [48]. We found a decreased level of MDM2 in miR-17-overexpressed cells (Fig 7b), but there was no change in p53 expression (Fig S3), which suggests that miR-17 may function in a p53-independent pathway. To confirm whether miR-17-3p targeted MDM2 directly, we generated three reporter constructs, each containing a fragment of wild-type or mutated MDM2 3'UTR sequence downstream of a luciferase coding sequence (Fig 7c). U87 cells were co-transfected with miR-17 plasmid and one of the constructs. There was a decrease in luciferase activities in the cells transfected with the MDM2 3'UTR construct, but the inhibitory effect was abolished when the miR-17-3p binding sites were mutated (Fig 7d).

**Figure 8:**
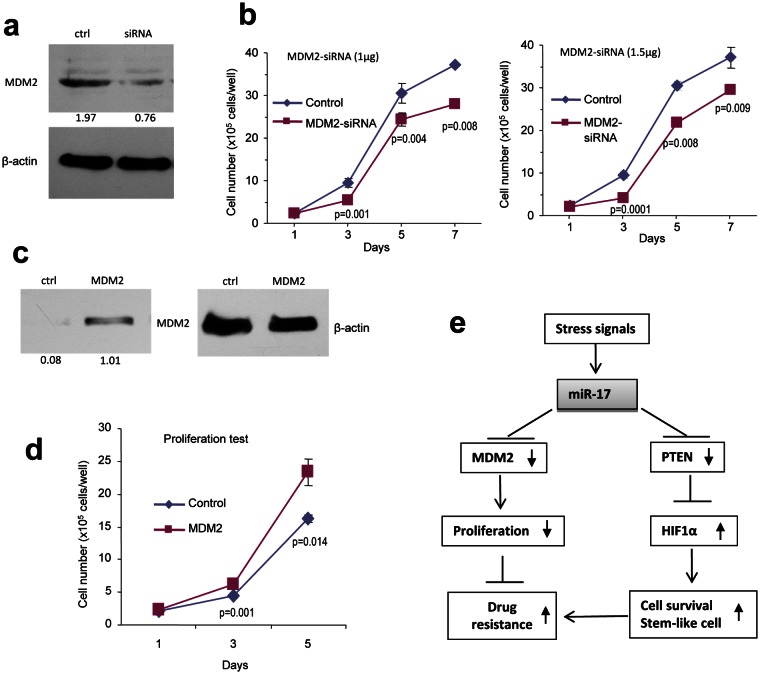
Confirmation of miR-17 functions by silencing and rescue assays (a) Cell lysates prepared from U87 cells transiently transfected with siRNA targeting MDM2 or a control oligo were subjected to Western blot analysis probed with anti-MDM2 antibody to confirm silencing of MDM2. (b) U87 cells transiently transfected with different amount of the siRNA or the control oligo were grown on 6-well tissue culture dishes. Cell proliferation was determined by counting the cells on day 1, 3, 5, and 7. * *p* < 0.01, **p<0.01. Error bars, SD (n = 3). (c) Cell lysate prepared from cells transiently transfected with MDM2 expression construct or the control vector and subject to Western blot analysis probed with anti-MDM2 antibody to confirm expression of the construct. Staining of β-actin from the same membrane confirmed equal loading. (d) U87 cells stably transfected with miR-17 were transiently transfected with MDM2 expression construct or the control vector and cultured for different days as indicated for proliferation assay. *p < 0.05. Error bars indicate SEM (n=4). (e) Proposed signal transduction showing the pathway by which miR-17 functioned.

We then validated whether MDM2 played an essential role in modulating U87 cell activities. The cells were transfected with siRNA complementary to MDM2. Silencing of MDM2 was confirmed using Western blotting (Fig 8a), and knockdown of MDM2 reduced cell proliferation (Fig 8b). To corroborate this result, we performed rescue experiments by transfecting U87 cells with MDM2 expression construct. After confirming up-regulation of MDM2 (Fig 8c), the effect of MDM2 on cell proliferation was tested, and we found that over-expression of MDM2 in the miR-17-transfected cells resulted in enhanced cell growth (Fig 8d).

## DISCUSSION

Given the fact that tumors often develop as a result of an aberrant response to a stress signal, it is important to determine the molecular biological mechanism involved. The theory of “tumor-starving therapy” suggests that tumor vascularization is critical to its survival. Therefore, bevacizumab, a monoclonal antibody against VEGF, has been approved to treat glioblastoma. It is believed to be able to starve tumors by blocking their blood supply. Nevertheless, highly penetrant tumor growth patterns in bevacizumab-treated patients have been repeatedly documented [49]. It is believed that a subpopulation of cells is resistant to “starving” treatment. Here we identify that miR-17-transfected glioblastoma cells survived longer under starved stress, with the potential to develop the tube-like structures of endothelial cells and to enrich GSCs. These may facilitate angiogenesis and increase the number of TSCs.

Our results revealed the unique response of HIF-1α to stimuli, suggesting a role of HIF-1α in mediating miR-17 functions. As a key transcription factor evoked upon exposure to hypoxia, HIF-1α can be observed best at a distance from blood vessels in tissue, but is absent immediately when oxygenated. In general, HIF-1α is ubiquitinated and degraded by VHL under normoxia but activated under hypoxia. In addition to that, it can be regulated by other suppressors such as PTEN and MDM2 [50]. PTEN's loss of function results in HIF-1α activation by dysregulation of the PI3K/AKT pathway, especially in glioblastoma cells [51]. The PTEN/PI3K/AKT pathway has been experimentally shown to be associated with stress adaptation, such as serum deprivation [52]. We showed that PTEN was one of the targets of miR-17 in glioblastoma cells. miR-17 could respond to stress signals by targeting PTEN, suggesting our findings might be of clinical implication. Firstly, because glioblastoma is characterized by its uncontrolled vascularization and high expression of miR-17 in tumor samples, miR-17 may take part in the process of glioblastoma angiogenesis by activating HIF-1α and VEGF indirectly. Secondly, since miR-17 endows cells with the ability to escape “tumor-starving therapy” by increasing survival and motility, cautions should be taken when treating patients with anti-angiogenesis therapy, especially for those who have tumors that are over-expressing miR-17.

We also demonstrated that miR-17 has dual roles in cell growth: it reduces the tumor proliferation rate, but protects cells from cytotoxic agents’ treatment. This is consistent with our previous data that elucidated that slower growing cells are more resistant to chemotherapy-induced cell death [53]. Currently, chemotherapeutic agents that are commonly used in treating glioblastoma act by interfering with DNA replication, such as temozolimide and carmustine. It is conceivable that fast growing tumor cells are more easily suffered from cytotoxic agents compared with slower growing cells. MiR-17 therefore can induce chemo-resistance on glioblastoma cells by slowing down their proliferation. In addition, a reduced cell proliferation rate also benefits cells under starved conditions because a slower metabolic rate requires limited nutritional supply. Thus, by targeting both MDM2 and PTEN simultaneously, miR-17 could act through several modes to regulate stress response. Very recently, similar finding were reported on other microRNAs (miR-141 and miR-200a) which potentially modulate the oxidative stress response in ovarian carcinogenesis [54]. It was concluded that miR-141 and miR-200a promoted tumor growth but sensitized tumors to chemotherapy, which was in agreement with our perspectives. These data support the emerging model of microRNA: a buffering function. This refers to a microRNA's ability to target several pathways as both a positive and a negative regulator [55]. Documented examples have shown that the buffering function of microRNA is critical to maintain homeostasis in the systemic network [33, 42]. It has been proposed that MDM2 can also ubiquitinate and degrade HIF-1α through the proteasome pathway, which raises the possibility that miR-17 mediates cellular response to diverse stimuli by targeting closely related signaling pathways [56].

The ability of miR-17 to induce generation of glioblastoma stem-like cells is another interesting finding of our work. Although the role of microRNAs in the development of TSC has been studied extensively, there is still no general agreement on the definitions of TSCs *in vitro* [57, 58]. It is known that TSCs can both undergo self-renewal and differentiate into a spectrum of mature cells. Moreover, recent discoveries indicate that they are widely involved in tumor progression, therapy resistance and distant metastasis. In glioblastoma, serum-free medium is a well-established method to enrich GSCs which can be detected by CD133 expression [59, 60]. Serum contains essential nutrition factors for tumor cell growth. During tumor progression to an advanced stage, it could be deprived of serum, under the stress of growth factor deficiency. In this study we reported that miR-17 not only increased CD133 positive cells when cultured in SFM, but also increased capacities of self-renewal and colony formation ability. This may be due to the activation of HIF-1α, which was documented to promote neurosphere formation in SFM [61]. To support this, we over-expressed HIF-1α in glioblastoma cells and measured the changes of GSCs. Not surprisingly, there was increased number of GSCs in HIF-1α-transfected cells compared with that of the control cells. Our findings confirm the critical role of HIF-1α in GSCs development and maintenance. More importantly, GSCs are often thought to be responsible for drug resistance, which may be another potential mechanism accounting for chemo-resistance in tumor cells over-expressing miR-17. At last, we found that miR-17 increased tumor cell migration and invasiveness, which can also be found in neural stem cells [60]. Taken together, miR-17 induced the generation of GSCs which display stem-like behaviors in multiple ways.

In summary, our findings reveal a novel mechanism of stress response in glioblastoma cells. During serum deprivation, miR-17 prolonged tumor cell survival, induced angiogenesis and promoted stem-like cell aggregation by repressing expression of MDM2 and PTEN and modulating HIF-1α levels. We thus proposed a signal pathway delineating miR-17 activities (Fig 8d). This adds new insights to our knowledge about microRNAs as mediators in tumor development. It has practical implications on clinical diagnosis and treatment. In glioblastoma patients, miR-17 could be used as a predictive marker of response to chemotherapy and anti-angiogenesis treatment. Although further studies are needed on the prognostic value of miR-17, our data suggests surgery and not drug treatment as a better option for patients who show over-expression of miR-17.

## MATERIALS AND METHODS

### Cell cultures

Human glioblastoma cell lines U87 (HTB-14) and U343 were cultured in DMEM media supplemented with 10% fetal bovine serum (FBS), penicillin (100 U/mL) and streptomycin (100 U/mL). Serum-free medium (SFM) was prepared by using DMEM-F12 medium supplemented with glucose (4.5 g/L), epidermal growth factor (EGF) (20 ng/mL) and fibroblast growth factor (FGF) (10 ng/ mL) [62]. Cells were maintained in a humidified incubator containing 5% CO_2_ at 37℃ and passed every 3-4 days as described [63].

### Construct generation

A cDNA sequence, containing two human pre-miR-17 units, a CMV promoter driving expression of GFP and an H1 promoter, was inserted into a mammalian expression vector pEGFP-N1 between the restriction enzyme sites *Bgl*II and *Hind*III [27, 64]. Green fluorescence was used to monitor transfected cells.

The primers’ sequences which were used in luciferase activity assay are listed in Supplementary Information Fig S5. The 3'-untranslated region (3'UTR) of MDM2 contains four potential binding sites for miR-17 while the 3'UTR of PTEN contains two. For each binding site, two pairs of primers were used to clone the fragments of 3'UTR and mutant controls. The PCR products were digested with *Sac*I and *Mlu*I, followed by insertion into a *Sac*I- and *Mlu*I-digested pMir-Report vector (Ambion) to obtain a luciferase construct or a mutant counterpart [65].

The PTEN cDNA with coding region was purchased from Origene and the HIF-1α is a generous gift from Dr. Peng at York University. The MDM2 cDNA was amplified by using two primers: MDM2-Kozak-*Bam*HI (5'cccggatccgccaccatgtgcaataccaacatgtctgtacc) and MDM2-CMyc-*Xba*l (5'ctatctagacaggtcctcctcggagatcagcttctgctccatggggaaataagttagcacaatcatttg). Then the PCR product was cloned into pCR3.1 vector (Invitrogen), and the identity of the insert was confirmed by DNA sequencing.

### RNA analysis

Total cell RNA was extracted using mirVana™ miRNA isolation kit (Ambion). RT-PCR was performed as described previously [66]. The primers specific for mature miR-17 were purchased from Qiagen. Human U6 RNA was used as control.

### Cell function test

In proliferation assay, transfected U87 and U343 cells were plated onto 100 mm tissue culture plates at a density of 1 × 10^5^ cells/well in DMEM containing 2.5%, 5% or 10% FBS and maintained for 5 days. Similarly, a survival assay was performed by starving the cells (1 × 10^6^ cells/well) in serum-free medium for up to 2 weeks. The cells were harvested and cell numbers were counted in different time points.

To test drug sensitivity, Docetaxel (Sanofi-aventis), Temozolomide (Merck) and Carmustine (Bristol-Myers Squibb) were applied to adhered cultures. All of the drugs were purchased from the Pharmacy Department at Sunnybrook Health Sciences Centre. The cell number was counted every other day after Trypan Blue staining.

### Cell migration assay

Migration studies were performed by wound scratch tests and transwell invasion tests respectively. In the scratch test, different serum concentration (0%, 2.5%, 5%, 10%) were applied in culture medium. The monolayer of cells was scraped linearly with pipette tips, washed to remove cell debris and replenished with fresh media. Microscope images of the scratch test were captured at the beginning and at different intervals later. In the transwell invasion assay, 24-transwell (Coster) was coated with 100 μl BD Matrigel™ (BD Biosciences) at a density of 1 mg/mL. 1 × 10^6^ of cells were suspended in DMEM containing 1% FBS and 100μL were transferred into the upper chamber of the transwell. The lower chamber was filled with 600 μL DMEM containing 10% FBS. After incubation for 24 hours, Matrigel™ was removed with a cotton swab and invaded cells were stained with Diff-Quick solution (Fisher Scientific).

### Tube formation assay

YPEN cells were mixed with miR-17-transfected cells or control cells and cultivated with BD Matrigel™ in 48-well plates. As described previously, the tube-like structures were observed and recorded by microscopic examination after 24 hours [67, 68].

### Western blot analysis

Western blot was performed as previously described [69].

### Flow cytometry

For cell cycle analysis, cells in the logarithmic phase were harvested and washed twice in PBS. Following adjustment of cell concentration to 2 × 10^6^ cells/mL in 50 μL PBS/HBSS with 2% calf serum, 1 mL 80% ice cold ethanol was added and incubated for 30 minutes. The cells were re-suspended in 500 μL HBSS containing 0.1 mg/mL of Propidium Iodide (Sigma) and 0.6% of NP-40. The DNA content was measured by flow cytometry (Beckman Coulter).

For antibody staining, 1 × 10^6^ cells were washed twice in PBS before re-suspension in 50 μL HBSS with 2% calf serum. Anti-CD133 antibody (Abcam, 1:20 dilution) was added and stained on ice for 30 minutes. The cells were pellet, and 1 μL of cy5-conjugated goat-anti-mouse (Jackson ImmunoResearch) was added into each tube. Flow cytometry analysis was conducted after 30 minutes and CD133 ratio was detected by FL4. The data was analyzed using FlowJo 9.1 software.

### Colony formation and self-renewal assay

Colony formation was assessed by mixing 1000 cells with 0.33% low-melting agarose in DMEM supplemented with 5% FBS and plated on 0.66% agarose-coated 6-well tissue culture plates. After four weeks, colonies were stained by Coomassie blue (Bio-Rad) and photographed. For cell self-renewal assay, cells were cultured in serum-free medium for two weeks before spheroid formed. Individual spheroids were plated at clonal density in non-adherent culture. Secondary spheroids were counted 5 days later.

### MTT assay

Ten thousand cells in 200 μL media per well were seeded and cultured in a 96-well plate for 24 hours. Two micro liters of drug in sequential diluted concentrations were added to each well and incubated overnight. Thiazolyl blue tetrazolium bromide (MTT) was diluted to 5 mg/mL in PBS and 20 μL were added to each well. After 3 hours, the cells were re-suspended in 200 μL of Dimethyl sulfoxide (DMSO) and shaken for 15 minutes. The absorbance value was recorded at 570 nm using a microplate reader (Perkin Elmer).

### Luciferase activity assay

Luciferase activity assays were performed as previously described [70]. In brief, U87 cells were seeded onto 12-well tissue culture dishes at a density of 1 × 10^5^ cells/well and co-transfected with the luciferase reporter constructs and miR-17 plasmid or positive control sequences with Lipofectamine 2000. After 12 hours, cell lysate was prepared by employing Dual-Luciferase® Reporter Assay Kit (Promega) and luciferase activity was detected using microplate scintillation and a luminescence counter (Perkin Elmer).

### Statistical analysis

All experiments were performed in triplicate and numerical data were subjected to independent sample t test (unless otherwise specified). The levels of significance were set at *p<0.05 and **p<0.01.

## Supplementary Figures


