# Supplementary material for: Stress Response of Glioblastoma Cells Mediated by miR-17-5p Targeting PTEN and the Passenger Strand miR-17-3p Targeting MDM2
Source: Oncotarget. 2012 Dec 31;3(12):1653–68. doi: 10.18632/oncotarget.810 (PMC3681502; doi:10.18632/oncotarget.810)
Supplement: Supplementary file 1 [file oncotarget-03-1653-s001.pdf]

Stress Response of Glioblastoma Cells Mediated by miR-17-5p Targeting PTEN and the Passenger Strand miR-17-3p Targeting MDM2

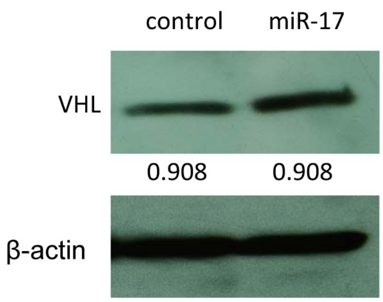

Fig S1. Western blot analysis of VHL expression

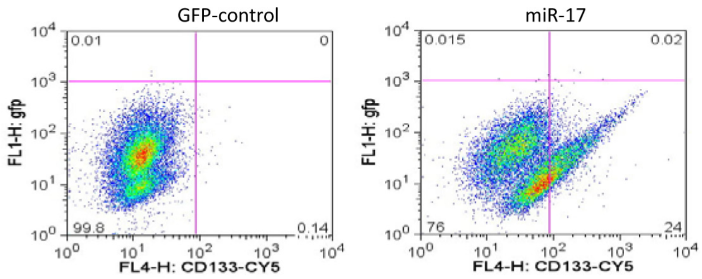

Fig S2. Flow cytometry analysis of CD133 expression

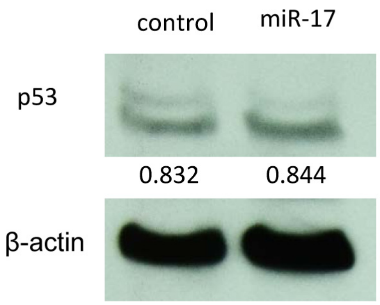

Fig S3. Western blot analysis of p53 expression

21-48.HuPten3084-R17-SacI	5’ gg **gagctc** agccttaccccgattcagcctcttcag
21-49.HuPten3084-R17-MluI	5’ cc **acgcgt** ttatt aag tgat gac taa ggct
21-50.HuPten3084-R17-MluI-mut	5’ cc **acgcgt** ttatt aag tgat gac taa ggctt **ttt cac** ctct
21-51.HuPten4252-R17-SacI	5’ gg **gagctc** gctagataatttgtggtacatctaaag
21-52.HuPten4252-R17-MluI	5’ cc **acgcgt** gagaa ttgt ga tgtt ata ttca
21-53.HuPten4252-R17-MluI-mut	5’ cc **acgcgt** gagaa ttgt ga tgtt ata ttca c **tttcac** ttac
23-14.huMdm2-mir17*a-SacI	5’ gg **gagctc** tagttgacctgtctataagagaat
23-15.huMdm2-mir17*a-MluI	5’ cc **acgcgt** gt gcg aaccc ggga ggc aga gc ttg
23-16.huMdm2-mir17*a-MluI-mut	5’ cc **acgcgt** gt gcg aaccc ggga ggc aga gc ttg gtc tga
23-17.huMdm2-mir17*b-SacI	5’ gg **gagctc** gtctccgcttcccgggttcaagcc
23-18.huMdm2-mir17*b-MluI	5’ cc **acgcgt** ctt gaa ccc tgtt ggc ggaa gttg
23-19.huMdm2-mir17*b-MluI-mut	5’ cc **acgcgt** ctt gaa ccc tgtt ggc ggaa gttg gtc tga
23-20.huMdm2-mir17*c-SacI	5’ gg **gagctc** tgggctagccaccgtaccacttgtc
23-21.huMdm2-mir17*c-MluI	5’ cc **acgcgt** gaa caca gcc agga ggc agag gttg
23-22.huMdm2-mir17*c-MluI-mut	5’ cc **acgcgt** gaa caca gcc agga ggc agag gttg gtc tga
23-23.huMdm2-mir17-SacI	5’ gg **gagctc** gagccgccacgcccagcctaataag
23-24.huMdm2-mir17-MluI	5’ cc **acgcgt** ttg tta cttc tg aaca cctt ccaa
23-25.huMdm2-mir17-MluI-mut	5’ cc **acgcgt** ttg tta cttc tg aaca cctt ccaa cac ctg

Fig S4. Primer sequences used to generate luciferase constructs

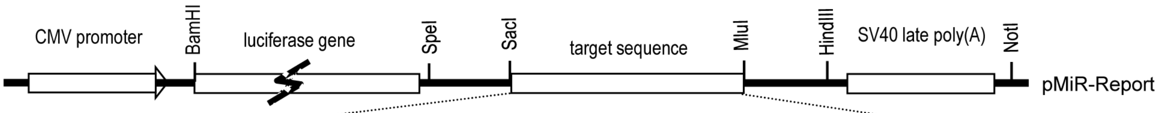

Luc-MDM2-1

GAGCTCtagttgacctgtctataagagaattatatatttctaactatataaccctaggaatttagacaacctgaaatttattcacatatatcaaagtgagaaaatgcctcaattcacata gatttcttctctttagtataattgacctactttggtagtggaatagtgaatacttactataatttgacttgaatatgtagctcatcctttacaccaactcctaattttaaataatttctactctgtcttaa atgagaagtacttggtttttttttttcttaaatatgtatatgacatttaaatgtaacttattattttttttgagaccgagtcttgctctgttacccaggctggagtgcagtggcgtgatcttggctcactg caagctctgcctcccgggttcgcac ACGCGT

Luc-MDM2-1mut

GAGCTCtagttgacctgtctataagagaattatatatttctaactatataaccctaggaatttagacaacctgaaatttattcacatatatcaaagtgagaaaatgcctcaattcacata gatttcttctctttagtataattgacctactttggtagtggaatagtgaatacttactataatttgacttgaatatgtagctcatcctttacaccaactcctaattttaaataatttctactctgtcttaa atgagaagtacttggtttttttttttcttaaatatgtatatgacatttaaatgtaacttattattttttttgagaccgagtcttgctctgttacccaggctggagtgcagtggcgtgatcttggctcaga ccaagctctgcctcccgggttcgcac ACGCGT

Luc-MDM2-2

GAGCTCgtctccgcttcccgggttcaagccattctcctggctcagcctctggagcagctgggattacaggcatgcaccaccatgcccagctaatttttgtatttttagtagagataggg tttcaccatgttggccaggctggtcacgaactcctgacctcaagtgaggtcacccgcctcggcctcccgaagtgctgggattgcagatgtgagccaccatgtccagccaagaattagt atttaaattttagatactcttttttttttttttttttttttttttgagacagagtcttgctccatcacccatgctagagtgcagtggagtgatctcggctcactgcaacttccgccttctgggttcaag ACGCGT

Luc-MDM2-2mut

GAGCTCgtctccgcttcccgggttcaagccattctcctggctcagcctctggagcagctgggattacaggcatgcaccaccatgcccagctaatttttgtatttttagtagagataggg tttcaccatgttggccaggctggtcacgaactcctgacctcaagtgaggtcacccgcctcggcctcccgaagtgctgggattgcagatgtgagccaccatgtccagccaagaattagt atttaaattttagatactcttttttttttttttttttttttttttgagacagagtcttgctccatcacccatgctagagtgcagtggagtgatctcggctcagaccaacttccgccttctgggttcaag ACGCGT

Luc-MDM2-3

GAGCTCtgggctagccaccgtaccacttgtcagcgtgaaaagtaagattgtaattgcctgtttagttttctgcctcatctttgaaagttccaccaagctgggaacctcttgattgtgagg cacaaatgtaagtacatcagaaaaaaacaaaaaaactggctttaaagcaggagcttgtgggcccctaagccagacggggactagcttttggcattatataattaagattttttaaatc cttaataagggttttattttatttttatttattttttgagacggagtcttgctctgtggctcaggctgga**gtacagtggtgcaatcttggctcactgcaac**ctctgcctcctggctgtgttc ACGCGT

Luc-MDM2-3mut

GAGCTCtgggctagccaccgtaccacttgtcagcgtgaaaagtaagattgtaattgcctgtttagttttctgcctcatctttgaaagttccaccaagctgggaacctcttgattgtgagg cacaaatgtaagtacatcagaaaaaaacaaaaaaactggctttaaagcaggagcttgtgggcccctaagccagacggggactagcttttggcattatataattaagattttttaaatc cttaataagggttttattttatttttatttattttttgagacggagtcttgctctgtggctcaggctgga**gtacagtggtgcaatcttggctcagaccaac**ctctgcctcctggctgtgttc ACGCGT

Luc-Pten-1

GAGCTCagccttaccccgattcagcctcttcagatactcttgtgctgtgcagcagtggctctgtgtgtaaatgctatgcactgaggatacacaaatatgacgtgtacaggataatgcct cataccaatcagatgtccatttgttactgtgtttgttaacaaccctttatctcttagtgttataaactccacttaaaactgattaaagtctcattcttgtcattgtgtgggtgttttattaaatgagag tatttataattcaaattgcttaaatccattaaaatgttcagtaatgggcagccacatatgattacaaagttcctgtgcatttttctatttttccccctccttgctatccttccaagcaaagcatcttt ctgtcatcttggtagacac**atacctgtctactcatggttaagaagagcacttta**agccttagtcatcacttaataa **ACGCGT**

Luc-Pten-1mut

GAGCTCagccttaccccgattcagcctcttcagatactcttgtgctgtgcagcagtggctctgtgtgtaaatgctatgcactgaggatacacaaatatgacgtgtacaggataatgcct cataccaatcagatgtccatttgttactgtgtttgttaacaaccctttatctcttagtgttataaactccacttaaaactgattaaagtctcattcttgtcattgtgtgggtgttttattaaatgagag tatttataattcaaattgcttaaatccattaaaatgttcagtaatgggcagccacatatgattacaaagttcctgtgcatttttctatttttccccctccttgctatccttccaagcaaagcatcttt ctgtcatcttggtagacac**atacctgtctactcatggttaagaagagGTGAAAa**agccttagtcatcacttaataa **ACGCGT**

Luc-Pten-2

GAGCTCgctagataatttgtggtacatctaaagcttagtcttaagtggcttgtgtgtggattttattcaacattcttgttgctagggtagagagaaatgttgctgagtagaaacaagagta cccagttcaatgtggtacagagagcagtccctaaaatctgtacacagtgtaatggaccactttaggagtcaagaggctgatttttcctatgaaattacattgcaacaggaagccttcta gtatagttccttttactgttagaatatgtttttatgcatacgctatagctgctttcccatcttccaacaacaggtatcaggatgtaagcaagctttaaacagtgtgaagatggcaggatagtgt catcggtaacagtcctctgactctaaa**tgtagttgctctgtaacactttg**tgaatataacatcacaattctc **ACGCGT**

Luc-Pten-2mut

GAGCTCgctagataatttgtggtacatctaaagcttagtcttaagtggcttgtgtgtggattttattcaacattcttgttgctagggtagagagaaatgttgctgagtagaaacaagagta cccagttcaatgtggtacagagagcagtccctaaaatctgtacacagtgtaatggaccactttaggagtcaagaggctgatttttcctatgaaattacattgcaacaggaagccttcta gtatagttccttttactgttagaatatgtttttatgcatacgctatagctgctttcccatcttccaacaacaggtatcaggatgtaagcaagctttaaacagtgtgaagatggcaggatagtgt catcggtaacagtcctctgactctaaa**tgtagttgctctgtaaGTGAAAg**tgaatataacatcacaattctc **ACGCGT**

Luc-Ctrl

actagtaatggagccacatgtatagatggcctcaatacatttacttgcctgtgtctaccaagctat ••••••••••••••• cattttatcccatgaggaacaagtcttcgtgaaccgtattgggcacgactaccagtggattggcctcaatgacaagatgtttgagcgtgatttc **gagctc**

Fig S5. sequences of the luciferase constructs.
